# Discovery of a Novel Specific Inhibitor Targeting Influenza A Virus Nucleoprotein with Pleiotropic Inhibitory Effects on Various Steps of the Viral Life Cycle

**DOI:** 10.1128/JVI.01432-20

**Published:** 2021-04-12

**Authors:** Fang Yang, Bo Pang, Kin Kui Lai, Nam Nam Cheung, Jun Dai, Weizhe Zhang, Jinxia Zhang, Kwok-Hung Chan, Honglin Chen, Kong-Hung Sze, Hongmin Zhang, Quan Hao, Dan Yang, Kwok-Yung Yuen, Richard Y. Kao

**Affiliations:** a Department of Microbiology, The University of Hong Kong, Hong Kong, China; b Research Center of Infection and Immunology, The University of Hong Kong, Hong Kong, China; c State Key Laboratory of Emerging Infectious Diseases, The University of Hong Kong, Hong Kong, China; d School of Biomedical Sciences, The University of Hong Kong, Hong Kong, China; e Department of Chemistry, The University of Hong Kong, Hong Kong, China; f Department of Biology, Guangdong Provincial Key Laboratory of Cell Microenvironment and Disease Research, Southern University of Science and Technology, Shenzhen, China; g Shenzhen Key Laboratory of Cell Microenvironment, Southern University of Science and Technology, Shenzhen, China; h SUSTech-HKU Joint Laboratories for Matrix Biology and Diseases, Southern University of Science and Technology, Shenzhen, China; The Peter Doherty Institute for Infection and Immunity

**Keywords:** influenza, nucleoprotein, small-molecule inhibitor

## Abstract

Influenza A viruses (IAVs) continue to pose an imminent threat to humans due to annual influenza epidemic outbreaks and episodic pandemics with high mortality rates. In this context, the suboptimal vaccine coverage and efficacy, coupled with recurrent events of viral resistance against a very limited antiviral portfolio, emphasize an urgent need for new additional prophylactic and therapeutic options, including new antiviral targets and drugs with new mechanisms of action to prevent and treat influenza virus infection. Here, we characterized a novel influenza A virus nucleoprotein (NP) inhibitor, FA-6005, that inhibited a broad spectrum of human pandemic and seasonal influenza A and B viruses *in vitro* and protects mice against lethal influenza A virus challenge. The small molecule FA-6005 targeted a conserved NP I41 domain and acted as a potentially broad, multimechanistic anti-influenza virus therapeutic since FA-6005 suppressed influenza virus replication and perturbed intracellular trafficking of viral ribonucleoproteins (vRNPs) from early to late stages. Cocrystal structures of the NP/FA-6005 complex reconciled well with concurrent mutational studies. This study provides the first line of direct evidence suggesting that the newly identified NP I41 pocket is an attractive target for drug development that inhibits multiple functions of NP. Our results also highlight FA-6005 as a promising candidate for further development as an antiviral drug for the treatment of IAV infection and provide chemical-level details for inhibitor optimization.

**IMPORTANCE** Current influenza antivirals have limitations with regard to their effectiveness and the potential emergence of resistance. Therefore, there is an urgent need for broad-spectrum inhibitors to address the considerable challenges posed by the rapid evolution of influenza viruses that limit the effectiveness of vaccines and lead to the emergence of antiviral drug resistance. Here, we identified a novel influenza A virus NP antagonist, FA-6005, with broad-spectrum efficacy against influenza viruses, and our study presents a comprehensive study of the mode of action of FA-6005 with the crystal structure of the compound in complex with NP. The influenza virus inhibitor holds promise as an urgently sought-after therapeutic option offering a mechanism of action complementary to existing antiviral drugs for the treatment of influenza virus infection and should further aid in the development of universal therapeutics.

## Abstract (precis)

Current influenza antivirals have limitations with regard to their effectiveness and the potential emergence of resistance. Therefore, there is an urgent need for broad-spectrum inhibitors to address the considerable challenges posed by the rapid evolution of influenza viruses that limit the effectiveness of vaccines and lead to the emergence of antiviral drug resistance.

## INTRODUCTION

Influenza A virus (IAV), which is the major cause of morbidity and mortality through both epidemic and pandemic influenza, remains a significant threat to public health and the global economy (1–4). Although vaccination remains the best strategy to prevent infection, available vaccines are effective against a limited number of viral strains due to the occurrence of mutations on viral RNA (vRNA) segments, the reassortment of vRNA segments between human and avian influenza virus strains, or the incorporation of new hemagglutinin (HA) subtypes. Thus, approved anti-influenza drugs, including M2 ion channel inhibitors (amantadine and rimantadine), neuraminidase inhibitors (NAIs) (oseltamivir and zanamivir), and a cap-dependent endonuclease inhibitor targeting the viral polymerase acidic (PA) protein (baloxavir marboxil), are the stand-alone treatments for infected individuals, especially during influenza pandemics without an effective vaccine (5–9). However, The CDC no longer recommends the M2 inhibitors for clinical use owing to drug resistance (6). Moreover, during the 2007–2008 influenza season, the circulating H1N1 strain gained nearly complete oseltamivir resistance (10). The quick spread of new influenza virus strains, cross-species transmission, and drug resistance (11) highlight an urgent need for new antiviral drugs (12) and combination therapies (13) with different mechanisms of action against alternative viral targets for the treatment of a broad spectrum of influenza viruses, particularly the recurring resistant strains and unexpected influenza pandemics.

IAVs have a segmented genome consisting of eight RNA molecules that are individually encapsidated into viral ribonucleoproteins (vRNPs), which represent the minimum set of proteins for viral transcription and replication (14, 15). Following increased insights into the importance of vRNPs, antiviral drugs that target nucleoprotein (NP) are an attractive strategy as NP is a highly conserved multifunctional protein that plays an essential role in infection by all subtypes of IAVs during the virus infection life cycle (16, 17). NP was reported to interact with RNA and oligomerize by self-interaction to form an oligomer to maintain the structure of RNP (18). In addition, NP is essential for viral transcription and replication; the major role of NP is the encapsidation of RNA into an RNP complex and maintenance of the conformation of the RNA template in a suitable order for transcription, replication, and assembly into virions (19). Another potential function of NP in transcription and replication is to switch the template mRNA transcription into vRNA replication (20). Furthermore, NP or RNPs participate in translocation between the cytoplasm and nucleus with the help of other viral proteins and host factors (21–24). Accumulating evidence suggested that NP was an ideal target for antiviral drugs due to its multifunctional role and high sequence conservation (25–28). Recent studies showed that viral replication is inhibited by small-molecule compounds that target NP, including nuclear localization signal (NLS)-binding compound mycalamide analogues (25) and NP salt bridge inhibitor compound 3 (26). Furthermore, our previous identification of nucleozin and its derivatives reported by others, which have been identified as NP inhibitors that trigger the aggregation of NP (28–30), prompted us to characterize other new potential drugs with the potential for elucidating their mode of action.

Previously, a chemical library of 50,240 compounds was screened using a high-throughput system (28). Here, we present one compound, FA-6005, out of 39 potential influenza virus inhibitors, as another NP inhibitor that impeded IAV infection with a low-micromolar median effective concentration (EC_50_) and protected mice from a lethal challenge dose of influenza virus H1N1 infection. We demonstrated that FA-6005 inhibits IAV replication via a mechanism that is different from those of existing antiviral drugs. The mode of action of FA-6005 was found to be interfering with the transcription and intracellular trafficking processes via interaction with NP. The crystal structure of the small molecule FA-6005 in complex with a soluble mimic of the NP trimer showed that FA-6005 coats the surface of NP, thereby inhibiting the multiple functions of vRNPs during virus infection and resulting in inhibitory effects on the entire viral life cycle. Taken together, the results of the current study showed that the newly identified NP pocket is a promising target for antiviral drugs that inhibit the multiple functions of NP. This detailed investigation also provides FA-6005 as a promising candidate for further development as an antiviral drug for the treatment of IAV infection and provides chemical-level details for inhibitor optimization.

## RESULTS

### Identification of a novel small-molecule inhibitor against influenza virus *in vitro* and *in vivo*.

**FIG 1:**
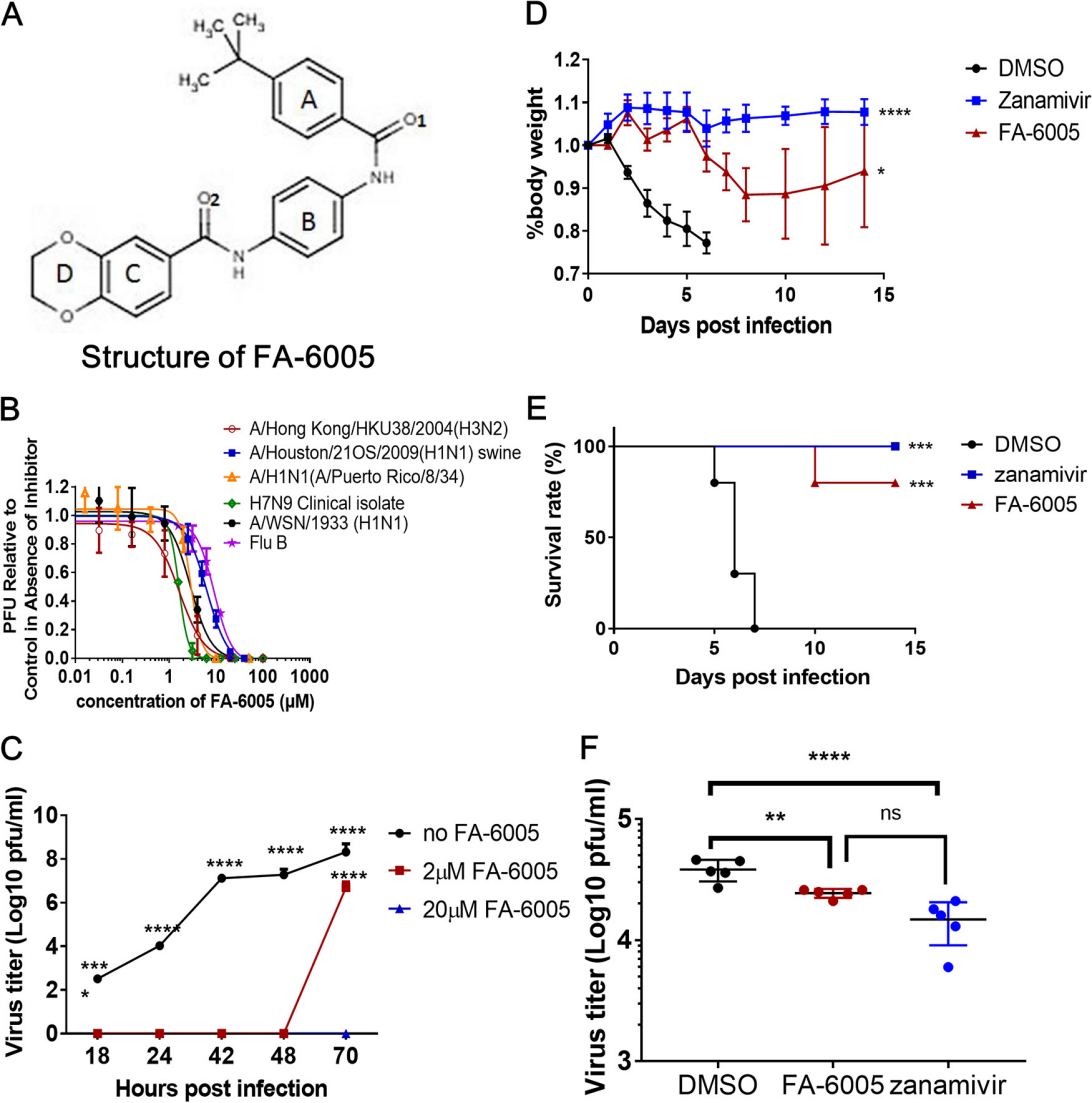
Chemical structure and antiviral activities of FA-6005. (A) Chemical structure of FA-6005. (B) Broad-spectrum antiviral activities of FA-6005 against influenza A virus subtypes and influenza B viruses. (C) Antiviral activity of FA-6005 in multicycle growth assays. (D to F) Efficacies of FA-6005 and zanamivir in a mouse influenza A PR/8/34 H1N1 virus infection model based on body weight (D), survival rate (E), and viral load in the lungs (F). Error bars represent the standard deviations (SD) of the means from three biological replicates (*n* = 3) in an individual experiment. Three independent experiments were performed for the PRA, viral growth assay, and animal study, and representative data are shown. *, results were significantly different from those of each control by a *t* test (*P*< 0.05); ****, results were significantly different from those of each control by a *t* test (*P* < 0.0001).

Based on the concept of chemical genetics, we have screened 50,240 structurally diverse compounds and selected and validated 39 potent antiviral compounds in our previous publication in detail (28). Upon evaluation of the antiviral activities and toxicities of the selected 39 hits, a novel compound, which we termed FA-6005, with potent antiviral activity on Madin-Darby canine kidney (MDCK) cells infected with influenza A/WSN/33 (H1N1) virus and a desirable selective index (SI) was selected for further characterization in this study (Fig. 1A). To assess its potential to inhibit virus replication, antiviral activities of FA-6005 against different strains of influenza A virus were determined by plaque reduction assays (PRAs). The results revealed that FA-6005 decreased the replication of all tested subtypes in a dose-dependent manner. FA-6005 impeded virus infection in MDCK cells by influenza A/WSN/33 (H1N1), A/Puerto Rico/8/34 (H1N1) (PR8), A/Hong Kong/HKU38/2004 (H3N2), A/Houston/21OS/2009 (H1N1), and A/Shanghai/02/2013 (H7N9) virus with EC_50_ (half-maximal effective concentration) values of 2.82 ± 0.33 μM, 2.88 ± 1.68 μM, 1.64 ± 0.31 μM, 6.22 ± 0.63 μM, and 1.61 ± 0.03 μM, respectively (Fig. 1B). Moreover, FA-6005 significantly suppressed viral growth at 2 μM and offered 100% protection of MDCK cells in the presence of 20 μM FA-6005 in a viral multicycle growth assay (Fig. 1C). The CC_50_ (50% cytotoxicity concentration) for FA-6005 was estimated to be 125.69 ± 0.61 μM, as illustrated in cell viability assay, resulting in an SI (CC_50_/EC_50_) as high as 43.6 against A/WSN/33 (H1N1) (data not shown). To demonstrate whether FA-6005 has broad-spectrum anti-influenza properties, the antiviral activity of FA-6005 against B/Wisconsin/01/2010 was evaluated. Most interestingly, we found that FA-6005 had similar antiviral activity against B/Wisconsin/01/2010, with an EC_50_ of 8.02 ± 0.81 μM (Fig. 1B). Thus, the above-described data demonstrated that FA-6005 had the capacity to inhibit the replication of both different subtypes of influenza A virus and influenza B viruses.

Since the free drug concentration in the tissue interstitial fluid is generally similar to the free plasma concentration under equilibrium, the doses of FA-6005 were selected according to the potential drug toxicity evaluated in mock-infected animals, maximum solubility in medium, as well as the existing data regarding the EC_99_ of FA-6005 *in vitro*. Therefore, BALB/c mice were inoculated with 10 50% lethal doses (LD_50_) of A/PR/8/34 (H1N1) and intraperitoneally (i.p.) injected with FA-6005 (20 mg/kg of body weight; final concentration of 100 μM per mouse) twice per day for 7 days, along with dimethyl sulfoxide (DMSO) and zanamivir (100 mg/kg) as controls. Sudden weight loss of all nontreated PR8-infected mice was observed during the first 5 days postinfection (p.i.), while the administration of FA-6005 or the potent NAI zanamivir resulted in a lower degree of weight loss, and the body weight was restored gradually compared to the control group (Fig. 1D). Moreover, DMSO-treated control mice showed severe morbidity and 100% mortality at day 7 postinfection. In contrast, zanamivir treatment apparently protected the mice from influenza virus-induced morbidity and lethality, while FA-6005 treatment resulted in a delayed time to death, with 80% surviving more than 14 days. The survival rates of PR8-infected mice treated with DMSO, FA-6005, and zanamivir were 0%, 80%, and 100%, respectively (Fig. 1E). In parallel, five mice from each group were euthanized on day 6 postinfection, and lungs were harvested to determine viral titers. The virus loads in the lungs of FA-6005 treated mice were significantly lower than those of the control group, while there were no significant differences compared to those of zanamivir-treated mice (Fig. 1F). Thus, these results collectively demonstrated that FA-6005 effectively inhibited both laboratory and clinical strains of both influenza A and B viruses *in vitro* and protected 80% of mice from death, suggesting that FA-6005 may be a promising drug against influenza viruses.

### Characterization of NP as the antiviral target of FA-6005.

**FIG 2:**
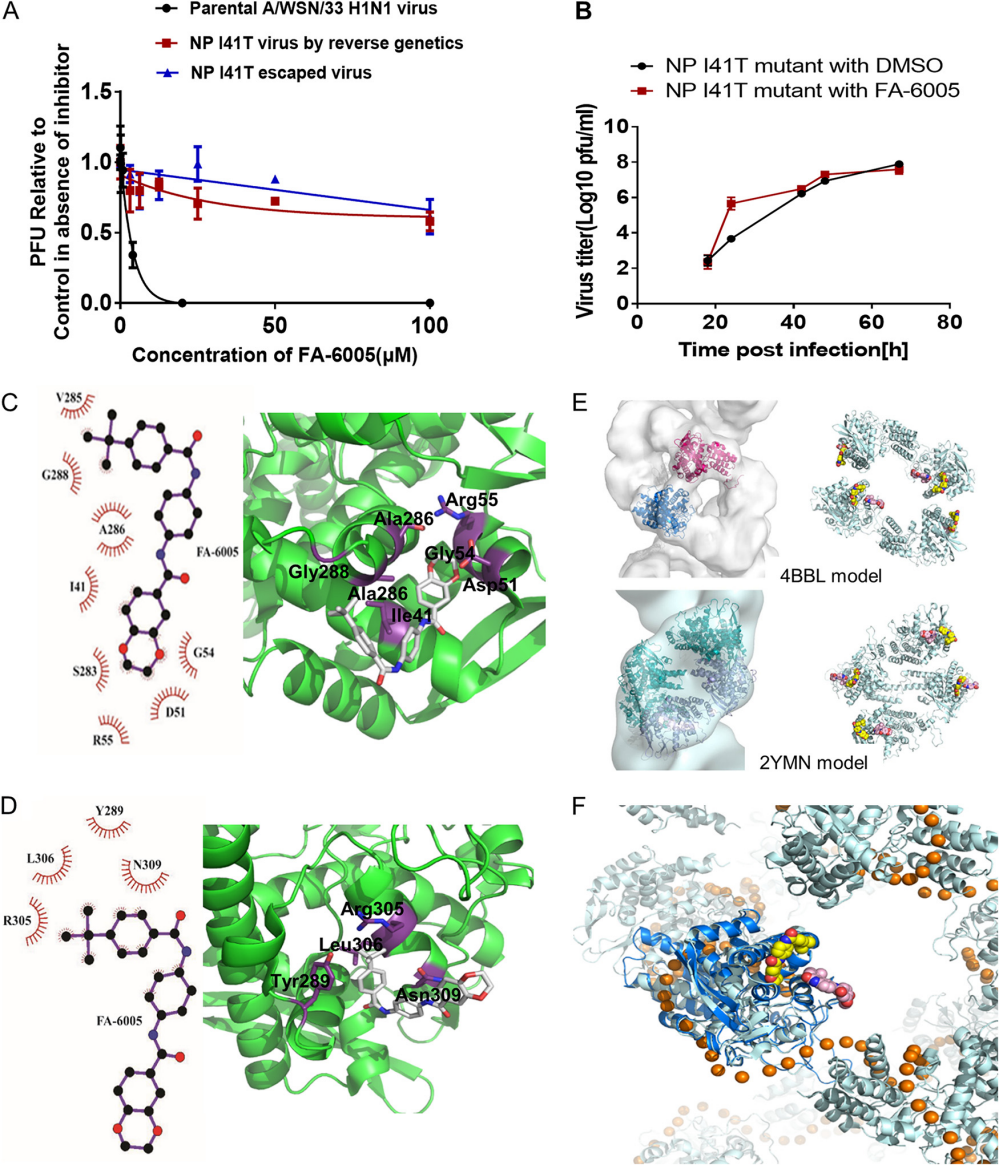
FA-6005 targets on influenza A virus NP. (A) Escape mutant virus and recombinant virus carrying the I41T substitution in influenza A virus NP confer resistance to high concentrations of FA-6005. (B) Growth kinetics of NP I41 mutant virus in the presence of FA-6005. (C) Crystal structure of the NP/FA-6005 complex showing the I41-binding pocket. (Left) The interacting residues of FA-6005 were determined by using LigPlot^+^ software (48). The compound exhibits hydrophobic interactions with I41, D51, G54, R55, S283, V285, A286, and G288. (Right) The binding pocket of FA-6005 on NP involves the I41 residue. The NP protein is in green, while the side chains of the interacting residues are shown in purple. (D) Crystal structure of the NP/FA-6005 complex showing the Y289-binding pocket. (Left) The interacting residues of FA-6005 were determined by using LigPlot^+^ software (1). The compound shows hydrophobic interactions with Y289, R305, L306, and N309. (Right) The binding pocket of FA-6005 on NP involves the Y289 residue. The NP protein is in green, while the side chains of the interacting residues are shown in purple. (E) RNP models and the possible FA-6005 positions on its central regions. Reconstruction of the central region of RNP was done using models under PDB accession numbers 4BBL and 2YMN, and ligands are fitted in the I41 and Y289 pockets. (F) Detailed view of modeled FA-6005 and RNA (represented by brown spheres) in one NP molecule.

To explore the target of FA-6005, we generated resistant mutant virus from A/WSN/33 (H1N1) by passaging the virus with increasing concentrations of FA-6005. The escaped mutant viruses resulting from 5 and 10 sequential passages were not susceptible to FA-6005 at concentrations higher than 100 μM (Fig. 2A), and the highly resistant mutants were used to identify the molecular targets of FA-6005. The whole genomes of both escape mutants and the wild-type (WT) virus were sequenced, and the corresponding amino acid changes in the mutants were summarized (data not shown). The EC_50_s of FA-6005 against the corresponding escape mutant viruses were higher than 50 μM (Fig. 2A). To further confirm that the resistance phenotype of mutant clones was attributable to these mutations, corresponding recombinant viruses were produced using reverse genetics (31). As demonstrated in the PRA, the recombinant NP I41T mutant virus showed resistance to high concentrations of FA-6005 and displayed a resistance profile similar to that of the originally isolated escape virus, while the other substitution mutations showed no resistance to FA-6005 (Fig. 2A and data not shown). The resistance-bearing mutation sites indicate that the target of FA-6005 is NP. Moreover, no significant differences were observed in viral replication kinetics of the NP I41T mutant virus in the absence or presence of 100 μM FA-6005 throughout the assay course, further supporting that FA-6005 may interact with NP (Fig. 2B). Furthermore, the growth kinetics of the NP I41T mutant virus was slightly lower than that of the wild-type virus prior to 45 h postinfection but eventually reached viral yields that were comparable to those of the wild-type virus (data not shown), indicating that the mutation in NP did not critically affect the fitness and infectivity of the recombinant virus.

To obtain structural insights into the interaction between NP and FA-6005, FA-6005 was cocrystallized with NP, and the complex structure was determined. Data collection and refinement statistics are summarized in Table 1. The NP/FA-6005 complex crystal belongs to space group C222_1_, with only one NP trimer in each asymmetric unit. Three FA-6005 molecules were found in each trimer. One was in the I41 pocket, and the major interactions involved in the I41 pocket included (i) hydrophobic interactions of the *t*-butyl group with G288, V285, and A286, ring C with I41, and ring D with G54 and R55; (ii) hydrogen bonding between amide O_2_ and T45; and (iii) dipole-dipole interactions of ring D with D51 and S283 (Fig. 2C). The second one was in the Y289 pocket, and the interactions involved in the Y289 pocket included hydrophobic interactions of the *t*-butyl group and ring A with Y289, R305, and L306 and rings A and B with N309 (Fig. 2D). The third one was around N224. However, N224 might not be a real pocket since the compound is seated between two symmetry-related NP molecules.

**TABLE 1:** Crystallographic data collection and refinement statistics

Statistic^*a*^	Value(s) for the NP/FA-6005 complex crystal^*b*^
Unit cell dimensions
*a*, *b*, *c* (Å)	119.60, 126.76, 194.43
α, β, γ (°)	90, 90, 90
Space group	C222_1_
Resolution range (Å)	43.50–2.80 (2.93–2.80)
Redundancy	6.5 (6.6)
Completeness (%)	92.3 (94.6)
*I*/σ	13.9 (2.6)
*R*_merge_ (%)	9.9 (62.2)
Refinement resolution range	43.50–2.8
No. of reflections	35,539
No. of protein chains	3
No. of ligands	3
*R*_work_	0.189
*R*_free_	0.255
RMSD (bond lengths) (Å)	0.01
RMSD (bond angles) (°)	1.648
Avg *B*-factors	48.5

a RMSD, root mean square deviation.

b Values in parentheses are for the highest-resolution shells.

To better characterize the binding of FA-6005 to NP, residues in the I41T and Y289 pockets were systematically mutated, and the effect of these point mutations on the inhibition of FA-6005 was measured by a PRA. We found that in the presence of FA-6005, mutation of residues in the I41 pocket displayed high resistance, while that of residues in the Y289 pocket exhibited more tolerance to the compound (Table 2). These mutation studies provided further evidence of two independent ligand-binding sites on NP. Since the resistance-bearing mutation sites of FA-6005 were mainly mapped to the I41 pocket, the scope of this project primarily focuses on an investigation of residue 41. Importantly, protein alignments indicated that residue 41 on NP is highly conserved among influenza A virus strains from different species, implying that influenza virus is less prone to developing resistance to FA-6005 (data not shown), and FA-6005 has broad-range anti-influenza activity (Fig. 1).

**TABLE 2:** Effect of selected NP point mutations on antiviral activities of FA-6005

NP residue	Mean EC_50_ (μM) of FA-6005 ± SD
Sensitive residues
I41T	>50
D51N	>50
A286T	>50

Tolerant residues
Y289H	9.33 ± 0.99
R305K	18.47 ± 1.17
S287A	10.98 ± 0.92
L306A	7.09 ± 1.06

Although Y289 was previously identified to be the binding site of nucleozin, which sits on either side of the putative I41-binding pocket, the binding sites of FA-6005 and nucleozin appear to be distinct, with a small amount of overlap. FA-6005 would bind on the external surface of the NP, and thus, it may not interfere with the interaction between NPs during oligomerization (Fig. 2E). Moreover, as illustrated in Fig. 2F, which shows the positions of FA-6005 and RNA, the binding of FA-6005 to NP molecules may not interfere with the interaction between NPs and RNA. Given the important role of the molecular target of FA-6005 in the virus life cycle and because FA-6005 coats the surface of the vRNPs, we therefore postulate that the compound-bound RNP may lose its ability to carry out proper biological functions, leading to inhibitory effects on the entire viral infection cycle.

### FA-6005 inhibits vRNP transcription and replication.

**FIG 3:**
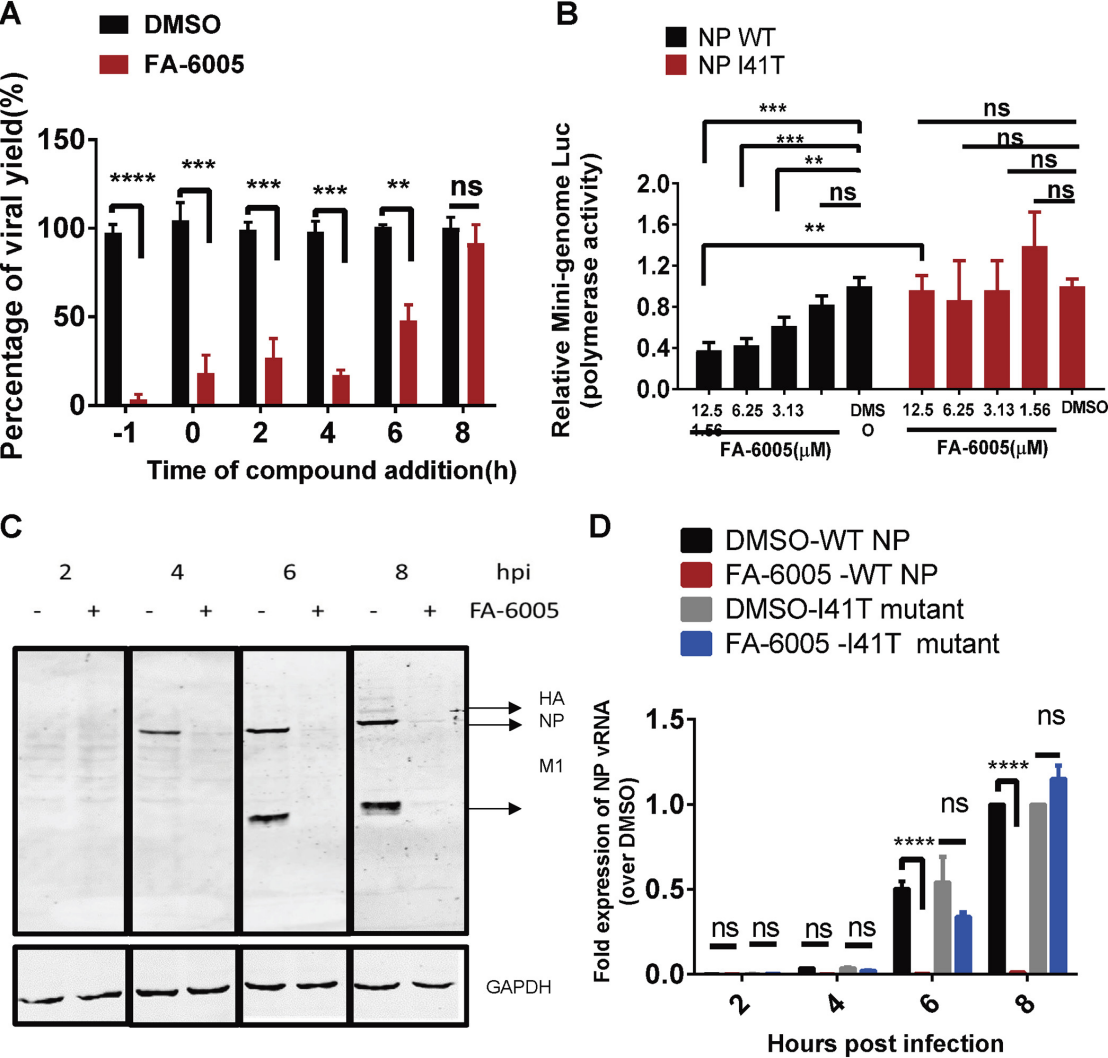
FA-6005 inhibits virus transcription and replication. (A) Time-of-addition experiments examining the effect of FA-6005 on various stages of the IAV life cycle. (B) FA-6005 exhibits inhibition of the parental virus NP activity but not the resistant I41T variant virus NP activity in a luciferase (Luc) reporter assay. (C) FA-6005 abolishes viral protein synthesis. MDCK cells were infected with IAVs at an MOI of 10 in the presence of 20 μM FA-6005. DMSO was added as a negative control. hpi, hours postinfection. (D) FA-6005 inhibits viral NP vRNA synthesis. **, results were significantly different from those of each control by Dunnett’s *t* test (*P < *0.005); ***, results were significantly different from those of each control by Dunnett’s *t* test (*P < *0.0005); ****, results were significantly different from those of each control by a *t* test (*P < *0.0001).

NP is the most abundantly expressed protein during influenza virus infection with multiple functions. NP plays a very important role in the viral life cycle, such as nuclear import of incoming vRNPs, replication, transcription, and nuclear export of newly produced viral genomes for packaging (16, 19, 32). Given that NP is the molecular target of FA-6005, it is speculated that FA-6005 exerts its inhibitory effect on stages that NP or vRNP is involved in, including virus replication and trafficking. To verify which stage of viral infection the compound was inhibiting, we conducted a modified PRA by adding the compound at different time points. Influenza virus was first inoculated with or without the addition of FA-6005 and incubated for 2 h for virus entry, and the unbound virus was then removed. An agarose overlay containing the corresponding concentration of the compound was added to the infected cells without FA-6005 treatment, while agarose with the DMSO control was overlaid onto the FA-6005-treated infected cells. The results showed that FA-6005 was able to maximally inhibit virus replication when it was added at 0 h p.i. or at the postentry stage of the viral life cycle (2 to 72 h p.i.) when it was added 2 h after infection. However, it is also suggested that FA-6005 interfered with virus entry and subsequently inhibited virus replication when it existed during the virus entry stage (0 to 2 h p.i.) but with a higher EC_50_ of 7.68 ± 0.50 μM (data not shown). To elucidate the mode of action of FA-6005, we therefore further determined the effects of varying the timing of the compound addition on the viral infection cycle by conducting a time-of-addition (TOA) experiment at a multiplicity of infection (MOI) of 1 (A/WSN/33) with FA-6005 treatments at −1, 0, 2, 4, 6, or 8 h relative to infection. Strong inhibition was indeed observed when FA-6005 was added 1 h before infection and up to 6 h after infection. However, FA-6005 did not inhibit virus replication when it was added 8 to 10 h after infection (Fig. 3A). Thus, these results indicated that FA-6005 interferes with various stages of the influenza virus life cycle, including the adsorption, entry, replication, transcription, and export processes.

Given the inhibitory effects exerted by FA-6005 during various stages of the virus life cycle and our characterization of the crystal complex of FA-6005/NP, we then postulated that the functions of NP or vRNPs during virus infection were restrained via the FA-6005/NP interaction and therefore that FA-6005 was sufficiently responsible for conferring antiviral activities. To examine this further, we first explored the possibility that FA-6005 affects viral genome replication and/or transcription, both of which occur during the 2- to 6-h-postinfection stages during the viral life cycle. In agreement with our results from the TOA assay, FA-6005 effectively abolished the replication of the virus in a dose-dependent manner in the modified luciferase reporter-based minigenome assay (33), which measures the polymerase activity of RNP in the context of a reconstituted viral ribonucleoprotein. However, FA-6005 had little effect on the NP I41T variant mutant, indicating that the I41T mutation overcame the observed antagonistic effects of FA-6005 against RNP polymerase activities *in vitro* (Fig. 3B). Thus, these results indicated that FA-6005 inhibits the activity of the vRNP complex.

To further validate the inhibitory effect of FA-6005 on the replication and/or transcription of IAVs, the macromolecular synthesis of viral proteins and RNAs was explored by quantitative PCR (qPCR) and Western blotting. The expression level of the viral proteins increased over time in the absence of the compound (Fig. 3C). Amounts of HA, NP, and M1 were hardly detected at 8 h postinfection when FA-6005 was added at the beginning of infection, indicating that FA-6005 had effectively abolished viral protein synthesis (Fig. 3C). By using strand-specific primers, the expression levels of vRNA, mRNA, and cRNA of NP and neuraminidase (NA) were examined (34). Increasing amounts of different types of viral RNA of both NP and NA (Fig. 3D and data not shown) were detectable from 4 h postinfection in the absence of FA-6005. Notably, viral RNA synthesis of both NP and NA was markedly reduced when FA-6005 was added, suggesting that the function of influenza virus polymerase activity was perturbed by FA-6005. The inability of FA-6005 to inhibit I41 variant NP in a minigenome assay (Fig. 3B) and the expression of vRNA (Fig. 3D) indicates that the I41T residue on NP is responsible for the resistance to FA-6005. Taken together, these results suggested that FA-6005 restrained the function of vRNP complexes during virus transcription and replication. We can also conclude that NP is the molecular target of FA-6005, with amino acid residue 41 as a critical binding partner.

### FA-6005 inhibits the nuclear export of NP or the vRNP complex.

**FIG 4:**
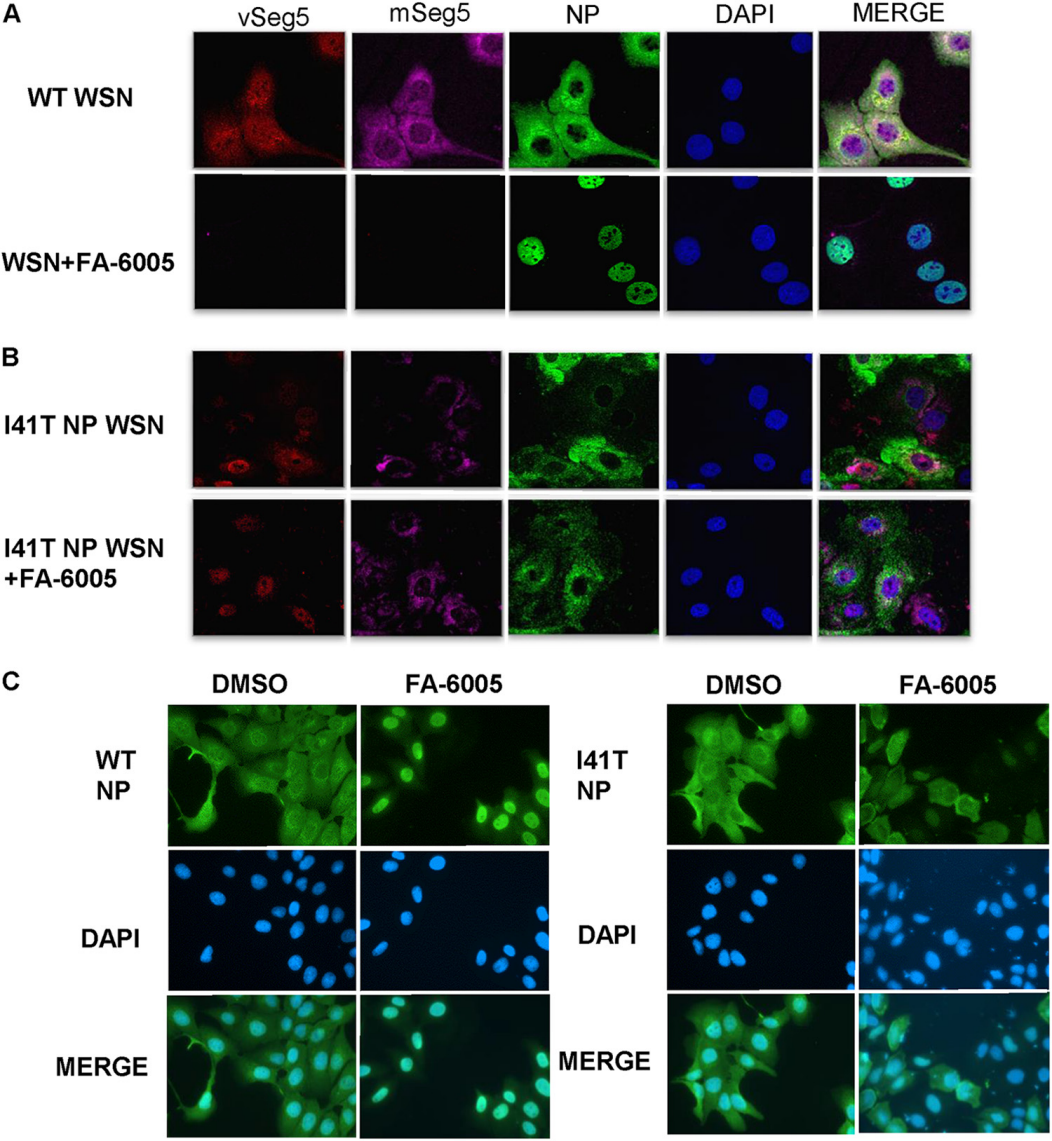
FA-6005 blocks vRNP and NP export. (A and B) FA-6005 kept WT vRNP retained in the nucleus (A), while it did not block I41T variant vRNP export (B). MDCK cells were first infected with A/WSN/33 (H1N1) virus or I41 mutant NP virus at an MOI of 10, and DMSO or 20 μM FA-6005 was added to each set at 2 h p.i. Samples were fixed at 10 h p.i., stained for NP (shown in green), and processed for FISH for the NP vRNAs (shown in red) and NP mRNAs (shown in purple). Merged images include a DAPI channel (shown in blue). (C) FA-6005 has an inhibitory effect on free NP export. MDCK cells were transfected with the phW2000-NP plasmid 24 h prior to the addition of compounds. Samples were fixed 8 h after DMSO or compound treatment and stained for NP (shown in green). Merged images include a DAPI channel (shown in blue). Each experiment was performed three independent times.

NP is the major component of the vRNP complex. Recent studies have demonstrated that blocking vRNP nuclear transport impairs the activity of the vRNP complex (19). Therefore, to further investigate the effects of FA-6005 on the translocation of the vRNP complex, fluorescence *in situ* hybridization (FISH) combined with immunoimaging was employed to visualize the migration of viral nucleoprotein and the distribution of vRNA and mRNA of segment 5 (NP) under the influence of inhibitors. NP/vRNPs in the cells treated with DMSO (negative control) were predominantly located in the cytoplasm at 10 h p.i., while FA-6005 treatment induced the retention of most of the NP/vRNPs in the nucleus (Fig. 4A), indicating that the nucleocytoplasmic transportation of the influenza vRNP complex was inhibited by FA-6005 during infection. Accordingly, reductions in viral vRNA and mRNA of segment NP were also detected, in agreement with our qPCR assay results (Fig. 4A). However, the nuclear export of the NP I41T mutant virus was not inhibited by FA-6005, further indicating that the NP-compound interaction results in the nuclear retention of NP/vRNPs (Fig. 4B). The influenza virus NP is a nuclear shuttle protein that contains both nuclear localization signals (NLSs) and nuclear export signals (NESs) (35). The I41T mutation is located in the putative NES area of NP (35), which raises the question of whether FA-6005 exerts effects on the localization of NP and thereby inhibits vRNP complex export. To test our hypothesis, we next examined the effects of FA-6005 on the translocation of NP in MDCK cells transiently expressing NP. Importantly, we found that NP was mainly detected in the cytoplasm at 24 h posttransfection (p.t.) in the control group, while WT NP export but not I41T NP export was retained in the presence of FA-6005 (Fig. 4C). Taken together, these results suggested that FA-6005 depressed NP/vRNPs export via interacting with residue I41 (located on a putative NES) of NP, thereby exerting an inhibitory effect on virus infection.

### FA-6005 perturbs the virus uncoating process and vRNP import at an early stage.

**FIG 5:**
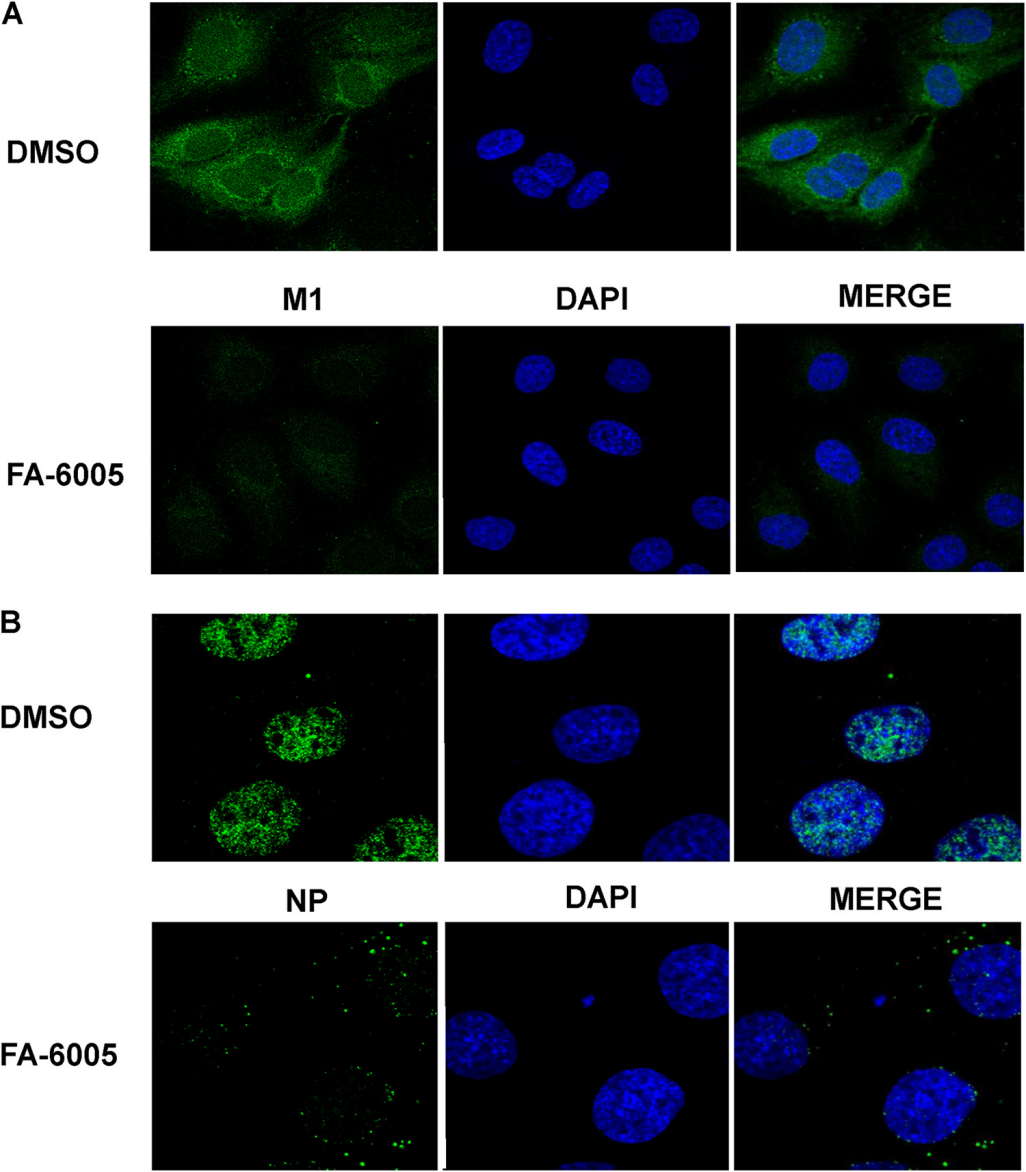
FA-6005 perturbs the entry step that NP is involved in. MDCK cells were infected with influenza A virus at an MOI of 100 in the presence of 1 mM cycloheximide. Infection was carried out on ice for 60 min to synchronize virus entry before incubation at 37°C for the times indicated. (A) The uncoating process was interfered in the presence of FA-6005. Disassociated M1 was visualized by M1 staining, and cell nuclei were visualized by DAPI staining. (B) Import of NP (green) was blocked in cells treated with FA-6005, while in the DMSO-treated cells, virus particles were allowed to enter the nucleus at 37°C for 3 h. Incoming NP proteins (green) were detected within the nucleus (blue) by staining with mouse monoclonal antibody. Each experiment was performed three times.

The results from the TOA assay indicated that FA-6005 impeded virus infection at the early stage of infection. To validate that the entry process perturbed by FA-6005 was not HA and NA specific, we explored the inhibitory activity of FA-6005 using pseudotype virus containing the luciferase gene and encoding either the vesicular stomatitis virus (VSV) envelope protein (pseudo-VSV) or H5 HA and N1 NA (pseudo-H5N1) in the presence of DMSO or FA-6005. The EC_50_s of FA-6005 against both pseudo-VSV and pseudo-H5N1 were found to be higher than 100 μM, further indicating that FA-6005 inhibits NP-involved entry steps (data not shown). pH-induced fusion of influenza virus envelopes with the limiting membrane of endocytic vacuoles provides the virus genome (that is, vRNPs) with a portal of access to the cytoplasm (32). Dissociation of vRNP from M1 is a prerequisite for vRNP translocation into the nucleus as it exposes the NLS (36). We therefore examined whether FA-6005 would affect the uncoating process that NP is involved in. As M1 disperses into the cytosol when undergoing pH-triggered disassociation from the vRNP complexes, M1 can be detected in the cytoplasm following the uncoating process (21). We then visualized M1 proteins to evaluate the dispersal level of M1 in the cytoplasm by an immunofluorescence (IF) assay. To prevent the synthesis of new viral proteins, 1 mM cycloheximide was added to the infection medium. Strong inhibition of the M1 signal was observed after the addition of FA-6005, indicating that FA-6005 inhibited the uncoating of virions and restricted the disassociation of vRNP-M1 complexes (Fig. 5A). Following uncoating, vRNPs are imported into the nucleus for viral transcription and replication. We next probed the action of FA-6005 during virus nuclear import. The nuclear accumulation of vRNPs was detected by an IF assay. Accordingly, the vRNPs failed to enter the nucleus in the FA-6005-treated group (Fig. 5B). Finally, trafficking of vRNPs at the early stage was perturbed by FA-6005. Therefore, these data collectively suggested that the inhibitory effect of FA-6005 at the early stage during virus infection resulted from NP or vRNPs, which further supports the conclusion that the molecular target of FA-6005 is NP.

### FA-6005 impairs the trafficking of circulating RNPs in the cytoplasm and leads to marked defects in virion budding.

**FIG 6:**
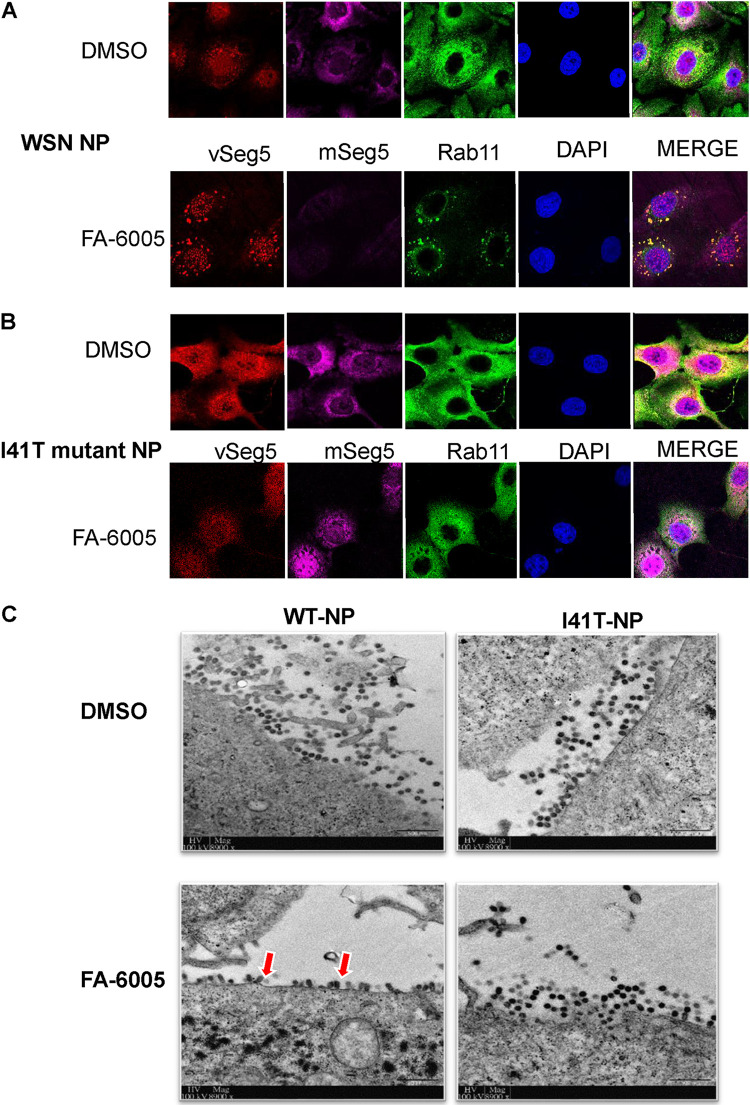
FA-6005 impairs vRNP cytoplasmic trafficking, leading to defects in virus budding. (A) FA-6005 induces WT vRNP and Rab11 aggregates in the cytoplasm. (B) I41T vRNP and Rab11 fail to aggregate in the presence of FA-6005. Samples were fixed at 8 h p.i., stained for NP (shown in green), and processed for FISH for the NP vRNAs (shown in red) and NP mRNAs (shown in purple). Merged images include a DAPI channel (shown in blue). (C) TEM visualization of budding virions in infected MDCK cells with or without drugs. MDCK cells were infected with WT or NP variant mutant viruses, treated with DMSO or FA-6005 at 6 h p.i., fixed at 8 h p.i., and processed for TEM. Images were acquired using an FEI Philips CM100 transmission electron microscope. Arrows indicate defective budding events. Each experiment was performed three independent times.

Our findings have indicated two mechanisms of action for FA-6005: the direct inhibition of viral transcription and the blocking of nuclear trafficking of NP. However, they could not explain why FA-6005 displayed a late-acting effect, as shown in the TOA assay (Fig. 3A). As FA-6005 has the same Y289-binding pocket as nucleozin, we thus postulated that FA-6005 might affect vRNP cytoplasmic trafficking to the apical plasma membrane (PM) region, a hallmark of late-stage infection, by trapping the vRNPs in the cytoplasm. We therefore infected MDCK cells with WT virus or the NP I41T mutant virus in order to monitor the localization of vRNPs by a FISH assay. Rab11 was also stained since Rab11-positive vesicles transport vRNPs to the PM region at the apical membrane (37). The majority of Rab11 was distributed throughout the cytoplasm in both treated and untreated cells. In the untreated cells, vRNA was exported from the nucleus at 6 h postinfection and accumulated in the cytoplasm at around 8 h postinfection, with a large portion of the vRNA and Rab11 being colocalized in the cytoplasm (Fig. 6A). In contrast, large RNP complexes colocalizing with Rab11 were seen in the perinuclear region in the FA-6005-treated group (Fig. 6A). Therefore, the timing of the effect that we observed, as well as the inclusion of Rab11 in the complexes (Rab11 interacts with RNPs but not non-RNP NP), suggested that the drug blocked the trafficking of RNPs. Importantly, the localization of vRNP and Rab11 exhibited no differences in the NP I41T mutant, with or without FA-6005 (Fig. 6B). Our results suggested that the interaction of FA-6005 with the vRNP-Rab11 complex perturbed cytoplasmic transport during the late stage of the infection cycle since the proper trafficking of the vRNPs for virion budding was halted.

The prominent cytoplasmic aggregates of Rab11 and vRNA clearly reflected the disruption of normal vRNP trafficking; hence, we next tested if this resulted from the drug “dismantling” assembled RNPs. A system in which cells are transfected with FLAG-NP and superinfected with virus to produce FLAG-tagged RNPs that can be affinity purified was enacted. A WSN/33 (H1N1) virus was inoculated into MDCK cells 12 h after FLAG-NP transfection, and cells were treated with the compound 6 h later and lysed at 8 h p.i. Western blotting of aliquots of the cell lysates and affinity fractionation over FLAG-magnetic beads were also examined. The results indicated that all of the viral proteins tested were present in the supernatant of infected cells in equal quantities, as was the cellular protein glyceraldehyde-3-phosphate dehydrogenase (GAPDH). The NPs in RNP and M1 were coprecipitated equally in the absence and presence of FA-6005 after affinity purification of FLAG-NP. Little effect was evident in the disruption of vRNP assembly by FA-6005 (data not shown). The system successfully detected RNP-M1 complex formation, which was not substantially changed by FA-6005 treatment. We also examined whether FA-6005 would influence the recruitment of RNA to NP by an electrophoretic mobility shift assay (EMSA). An RNA shift pattern was visualized by staining with ethidium bromide (EtBr), while the protein display pattern was stained by Coomassie brilliant blue G-250. From the gel staining, it was discovered that NP-RNA-binding activity, as well as NP-RNA complex formation, did not change with or without FA-6005 (data not shown), which is consistent with our NP/FA-6005 cocrystal structure. Therefore, it can be concluded that the FA-6005-induced aggregation of vRNPs in the cytoplasm did not result from the disruption of RNP assembly and the NP-vRNA interaction. These results are consistent with our speculation for the NP/FA-6005 crystal complex.

vRNP cytoplasmic trafficking is interconnected in an intricate network whereby disruption of one pathway can have secondary effects on other vesicular pathways. To eliminate the possibility that FA-6005 may exert a general effect on the exocytic pathway and, as a consequence, induces disruption of the trafficking of viral membrane proteins, we then examined the localization of α-tubulin, which participated in the RNP-Rab11 transport pathway, and the distribution of HA was also investigated by a FISH assay. The localizations of both α-tubulin and HA were found to be identical between the untreated and treated groups, while compound-induced aggregation of vRNP was observed (data not shown). Thus, the normal trafficking of cellular and additional viral proteins suggested that FA-6005 did not affect the function of the microtubule network, and the effects of FA-6005 on viral and cellular trafficking pathways are apparently specific to the RNP-Rab11 complex in infected cells.

As a consequence of impaired RNP cytoplasmic trafficking, the viral genome would be impeded, reaching the PM in the late stage of the virus life cycle, leading to markedly defective budding of virions. To investigate this relationship, we analyzed virions budding from WT and variant mutant viruses in the absence or presence of FA-6005 by transmission electron microscopy (TEM). As illustrated in Fig. 6C, mature viruses budded out from the PM by 8 h p.i. in the control group, while few mature virions were detected after treatment with FA-6005 from 6 h p.i. onward, and the budding event exhibited obvious defects compared to the untreated group. Importantly, no significant difference was observed regarding the budding process in NP I41T mutant virus with or without FA-6005. Thus, FA-6005 clearly impeded vRNP cytoplasmic trafficking and resulted in virion budding defects, which can match the antiviral effects of FA-6005 in the late stage of viral infection (Fig. 3A).

Collectively, we have successfully identified another novel NP inhibitor, FA-6005, with a mode of action distinct from those of previously reported inhibitors. The cocrystal structure of the NP/FA-6005 complex clearly showed the binding pocket of FA-6005 and suggested potential mechanisms. We have also elucidated the inhibitory mechanism of FA-6005 and showed that it exerts pleiotropic inhibitory effects on various steps of the viral infection cycle. FA-6005 inhibited viral RNA and protein synthesis and the postentry process, including uncoating, nuclear import, and export processes, when FA-6005 was present at the start of the infection. Moreover, when added at later time points, FA-6005 potently blocked virus infection without affecting viral macromolecular synthesis. Instead, it induced large perinuclear aggregates of RNPs with cellular Rab11 that had undergone nuclear export, thereby leading to much-reduced production of virus progeny. Furthermore, the amino acid residues found in the binding pocket at FA-6005 are highly conserved among strains of influenza virus, suggesting that the small pocket within NP may be a promising target for antiviral drugs, particularly a multifunctional NP inhibitor. This work also provides proof that FA-6005 is a new lead anti-influenza virus compound and highlights a novel strategy for developing new drugs that target the NP pocket.

## DISCUSSION

Influenza virus poses a global public health concern and remains a human menace that has not been fully addressed yet. Currently, we are limited in the countermeasures to prevent and treat influenza virus infection. Influenza virus claims 250,000 to 500,000 lives annually, even with the availability and use of vaccines and limited antiviral drugs (38, 39). While M2 ion channel inhibitors are not recommended for clinical use due to their reduced effectiveness, NAIs and a recently approved cap-dependent endonuclease inhibitor, baloxavir, are the mainstays of antiviral treatment for influenza virus infections (9). However, with regard to the segmented reassortment features, coupled with the vast range of natural hosts for influenza A virus and the emergence of viruses with reduced susceptibilities to NAIs and baloxavir, novel antiviral drugs with different mechanisms of action are therefore urgently needed.

Influenza virus nucleoprotein stands out as one of the most promising drug targets since it is highly conserved among influenza virus strains isolated from different species (16). The multifunctional role of NP provides a number of opportunities for therapeutic intervention. This study has successfully illustrated that chemical genetics is an effective approach and a powerful tool to identify novel small-molecular inhibitors against influenza virus. Following the high-throughput screening of 50,240 compounds, we identified a novel compound, FA-6005, as a potential anti-influenza virus NP inhibitor with a defined mechanism of action. FA-6005 was shown to extend its breadth to multiple influenza A virus subtypes and even to influenza B viruses. Furthermore, *in vivo* data indicated that FA-6005 prevented body weight loss, increased the survival rate, and reduced the viral titers of influenza virus-infected mice, thereby rendering it a promising lead compound for the treatment of severe influenza (Fig. 1). Although FA-6005 showed less efficacy than zanamivir for the treatment of influenza A virus-infected mice, the inhibitory effect of this compound could be further improved by the development of its congeners. Moreover, it has been reported that emergent amino acid substitutions in the polymerase acidic (PA) protein occurred at a frequency of 2 to 20% and were found to confer reduced susceptibility to baloxavir (40). The rise in resistance to currently approved influenza antivirals such as amantadine has emphasized the potential benefits of antiviral cocktails of small molecules for future therapeutic strategies (13). Given the different target of FA-6005, this compound would be a good candidate to include in a potential anti-influenza drug cocktail by providing synergistic effects combined with the current clinical use of zanamivir or baloxavir for reduced-susceptibility virus treatment and lowering the chances of developing resistance.

Substitution mutation I41 was discovered in the viral nucleoprotein for resistant mutants raised with FA-6005, as resistant phenotypes were confirmed by reverse genetics. X-ray crystallography incorporating FA-6005 and monomeric NP identified two potential binding sites for FA-6005 (Fig. 2). Consistent with our mutational study, FA-6005 interacts with several amino acid residues around the I41-binding pocket: I41, D51, and A286, etc. Furthermore, the configuration of FA-6005 at the NP I41 pocket also revealed strong binding energy in our molecular docking model (data not shown). Moreover, the residues around the Y289-binding pocket are less resistant to FA-6005, indicating that the Y289 pocket is not the major binding domain for FA-6005. Taken together, the results show that FA-6005 may exert inhibitory effects against IAVs through interaction with residues at the I41 pocket, and the crystal structures were also very helpful in rationalizing the mechanisms of action of FA-6005.

NP is highly conserved at the FA-6005-binding pocket across seasonal, pandemic, and highly pathogenic avian influenza A viruses. NP I41 is highly conserved in currently circulating influenza viruses, as the most common amino acid at position 41 in the NP is isoleucine (99.8%), and the frequency of the polymorphic natural mutation NP I41V in 2,223 human influenza A virus strains was only 0.2%. Therefore, the resistant mutation NP I41T generated by FA-6005 perhaps occurs only in the presence of our compound. Thus, it is not yet obvious that the resistance of viruses to FA-6005 would be problematic during clinical use.

The broad-spectrum features of FA-6005 against various influenza A virus strains could be explained as amino acids that are well conserved among influenza A virus species, although their functional importance for viral replication remains to be further studied. However, it is surprising that FA-6005 also has its inhibitory properties against influenza B virus, with an EC_50_ of 8.02 ± 0.81 μM, whose structure is quite different from that of influenza A virus. The NPs of influenza A and B viruses do not share identical amino acids at the identified resistance mutation sites I41 and R55 but show identical amino acids at position D51. By mapping the corresponding resistance mutations onto the crystal structure of the influenza B virus NP, it was found that I41 was located on the surface and will not form a pocket for FA-6005 to access. These results suggest that FA-6005 may interact with influenza B virus NP at a different position, which is worthy of further study.

FA-6005 was found to perturb virus transcription and replication at a postentry stage, while it was also found to inhibit virus entry and subsequently inhibit virus infection but with a higher EC_50_ in a modified PRA (data not shown). Such results indicated that FA-6005 interferes with various stages of the influenza virus life cycle, including the adsorption, entry, replication, transcription, and export processes. Moreover, FA-6005 showed distinct inhibitory effects on influenza virus infection in a TOA assay (Fig. 3), indicating that FA-6005 inhibited viral replication via a pathway different from those of approved antiviral drugs. The mode of action of FA-6005 in infected cells was further demonstrated by the isolation and characterization of NP I41T variants with reduced sensitivity to the compound. When added early until the end of one cycle during virus infection, FA-6005 was found to inhibit RNP activities and viral RNA and protein synthesis. However, this cannot explain the early-acting drug effect when FA-6005 is present at 0 to 2 h p.i. during the virus life cycle. Further analyses showed that FA-6005 interfered with virus entry involving NPs, including the uncoating process and the nuclear import process. Furthermore, we found that FA-6005 also blocked the nuclear export of vRNPs, which may be because residue I41 is predicted to be located in a putative NES (35). The above-described results indicate that the NP/FA-6005-binding pocket may play a role in M1-RNP disassociation and vRNP shuttling between the nucleus and cytoplasm.

Given that no effect on viral RNA and viral protein synthesis but induced rapid aggregation of cytoplasmic RNPs with cellular Rab11 was observed when the compound was added during the late stage of virus replication, such aggregation of the RNP-Rab11 complex also correlated with a marked reduction in the amount of virus budding. More importantly, we saw little effect of FA-6005 treatment on other viral proteins and microtubule networks during the cytoplasmic trafficking stage. We therefore conclude that the late-acting effect of FA-6005 on virus replication is directly attributable to effects on RNP cytoplasmic trafficking, and the proper trafficking of the viral genome may be specifically required to allow efficient virus assembly and/or budding. Taken together, the results described above could be due to the protein aggregation capacity of FA-6005 as the location of the Y289-binding sites of FA-6005 overlaps those conferring resistance to nucleozin, whose mechanism is defined to cause aggregation. These results expand the role of the interacting binding pocket in NP nuclear transport and virus replication and strongly support a distinct antiviral mechanism of FA-6005 in prohibiting virus replication and trafficking.

Recently, several compounds targeting NP have been identified as novel antiviral drug candidates (26, 27, 41–43). However, they showed weak antiviral effects since they disrupt only one of the NP functions such as NP export or self-oligomerization and RNA-binding capacities. Developing a drug that inhibits multiple NP functions will be extremely effective because NP is the most abundant multifunctional viral protein in infected cells (16). While the mechanism of nucleozin is well defined (28, 30), the rapid appearance of resistant viruses and the limited antiviral spectrum of nucleozin prompted us to characterize other NP inhibitors that perturbed several NP functions. Our current study reveals an important antiviral mechanism of FA-6005 by targeting influenza A virus nucleoprotein with pleiotropic inhibitory effects on various steps of the viral life cycle. We also provide evidence that the binding pocket residues of FA-6005 are effective druggable targets by structural analysis and identify its multifunctional role during virus infection.

Given the different targets between currently approved anti-influenza therapeutics and our compound, it is expected that the NP I41T variant mutant virus is highly susceptible. It has also been reported that a single use of baloxavir is associated with the frequent emergence of variants with reduced susceptibility (40). However, the use of a combination of antiviral compounds that could rapidly reduce viral replication and limit the emergence of antiviral-resistant strains remains an attractive approach. Thus, it is highly promising that the current generation of the NP inhibitor FA-6005, particularly when used in combination with currently approved NAIs or baloxavir, has the potential to be added to our therapeutic armamentarium in fighting against the fast-evolving influenza virus and providing our repertoire of antiviral options with more effective treatments for severely ill or hospitalized patients. Moreover, it has been reported that single-dose baloxavir showed significant postexposure prophylactic efficacy in preventing influenza in household contacts of patients with influenza (44); thus, considering FA-6005 as another broad-spectrum inhibitor with pleiotropic inhibitory effects on the functions of vRNPs of influenza virus, it is highly anticipated that our current compound can be considered both prophylaxis and therapy for influenza A and B virus infections.

## MATERIALS AND METHODS

### Cell lines and viruses.

293T and Madin-Darby canine kidney (MDCK) cell lines were purchased from the ATCC and shown to be mycoplasma free using the MycoAlert mycoplasma detection kit (catalogue number LT07-318; Lonza). Cells were maintained in minimal essential medium (MEM) or Dulbecco’s modified Eagle’s medium (DMEM) with 10% heat-inactivated fetal bovine serum (HI-FBS). Influenza A virus subtypes A/WSN/33 (H1N1) and A/PR/8/34 (H1N1) were propagated in MDCK cell cultures in plain MEM supplemented with 0.2% FBS. Other virus strains, including A/Hong Kong/415742/2009 (H1N1), A/California/NHRC0007/2005 (H3N2), a clinical isolate (H7N9), and B/Wisconsin/01/2010, were propagated in either MEM or DMEM without FBS. All experiments involving the live clinical isolate (H7N9) were performed according to standard operating procedures of the approved biosafety level 3 facility as described in a previous report (45).

### Antibodies.

Influenza A virus nucleoprotein antibody (catalogue number MAB8257; EMD Millipore), GAPDH antibody (catalogue number G9545; Sigma-Aldrich Inc.), influenza A virus M1 antibody (catalogue number GTX125928; Genetex), anti-influenza A virus antibody (catalogue number ab1074; EMD Millipore), influenza A virus H1N1 HA (hemagglutinin) antibody (catalogue number GTX127357; Genetex), α-tubulin monoclonal antibody (catalogue number A11126; Thermo Fisher Scientific), Rab11 polyclonal antibody (catalogue number 71-5300; Thermo Fisher Scientific), donkey anti-mouse IgG(H+L) secondary antibody Alexa Fluor 488 (AF488) (catalogue number A32723; Thermo Fisher Scientific), donkey anti-mouse IgG(H+L) secondary antibody AF594 (catalogue number A-11032; Thermo Fisher Scientific), and horseradish peroxidase (HRP)-linked goat anti-rabbit antibody (catalogue number 31460; Thermo Fisher Scientific) were used.

### Plaque reduction assay.

The plaque reduction assay (PRA) was performed in triplicate as described in detail previously (28). In brief, confluent MDCK cells were seeded in 24-well tissue culture plates (TPP, Switzerland) using MEM (Thermo Fisher Scientific, USA) in 10% FBS 1 day prior to conducting the assay. After 16 to 24 h, cells were infected with 50 PFU of influenza virus for 24-well plates with or without the addition of serially diluted compounds. Infected cells were incubated at 37°C with 5% CO_2_ for 1.5 h before removing unbound viral particles by aspiration. At the end of incubation, MEM was mixed with 1% FBS, 1 μg/ml tosylsulfonyl phenylalanyl chloromethyl ketone (TPCK)-treated trypsin (Thermo Fisher Scientific, USA) containing the corresponding concentrations of compounds, and 0.75% low-melting-point agarose (LMA) (Thermo Fisher Scientific, USA) and overlaid onto the infected cells. Agarose plugs were then removed, monolayers were stained with 0.7% crystal violet (BDH, Poole, England), and the plaques were counted. The percentage of inhibition of plaque formation at each compound concentration relative to the control (without the compound) was determined, and the median effective concentration (EC_50_), representing the concentration of a compound that is required for 50% inhibition *in vitro*, was calculated by Sigma plot (SPSS, USA). No FBS was included in the overlying medium for strains A/Hong Kong/415742/2009 (H1N1) and A/California/NHRC0007/2005 (H3N2), the clinical isolate (H7N9), and influenza B viruses.

### Cell viability assay.

A CellTiter-Glo kit (Promega) was used to test the cell viability of selected compounds by the detection of ATP levels as a function of cell viability according to the manufacturer’s instructions. The assay was performed by seeding MDCK cells at 20,000 cells/well in a total volume of 100 μl MEM with 10% FBS to 96-well cell culture plates 1 day prior to conducting the assay. After 24 h, cells were washed once with 1× phosphate-buffered saline (PBS) and replenished with MEM before the addition of the compound. The compound was serially diluted in 96-well plates using MEM with 1% FBS, starting from 1 mM, by 2-fold dilution. The monolayer MDCK cells were treated with serially diluted FA-6005 or dimethyl sulfoxide (DMSO) as the control in triplicates. After 72 h of incubation at 37°C with 5% CO_2_, a volume of CellTiter-Glo reagent equal to the volume of the cell culture medium present in each well was added. The luminescence signal was read using a DTX880 multimode detector, followed by incubation of the reagent and cells for 10 min at room temperature (RT). The relative metabolic activity of ATP was calculated by normalization of the mean raw signals for each concentration of the compound to the mean signal for the negative control. This calculation normalizes the negative control to 100% ATP activity.

### Animal experiments.

Five- to seven-week-old BALB/c female mice were kept in biosafety level 2 housing and provided access to standard pellet feed and water *ad libitum*. All experimental protocols were performed according to the standard operating procedures of approved biosafety level 2 animal facilities and were approved by the Committee on the Use of Live Animals in Teaching and Research (CULATR). Experiments were performed in triplicate as reported previously (28). As the maximum solubility threshold of FA-6005 is 100 μM in 2% DMSO–MEM using a generic ultraperformance liquid chromatography (UPLC) method, we therefore first dissolved FA-6005 in DMSO to prepare a 250 mM stock solution. Thirty-microliter stock solutions containing FA-6005 or DMSO were further dissolved in 3 ml 50% (vol/vol) methylcellulose–PBS to make 2.5 mM solutions. The doses of candidate molecule FA-6005 were selected according to the potential drug toxicity evaluated in mock-infected animals. Moreover, since the free drug concentration in the tissue interstitial fluid is generally similar to the free plasma concentration under equilibrium, we can thus link the EC_99_ of FA-6005 *in vitro* to its *in vivo* tissue interstitial fluid concentration. Therefore, the mice were divided into three groups: one group (10 mice) was injected intraperitoneally (i.p.) with zanamivir (100 mg/kg) (GlaxoSmithKline), a second group (10 mice) was injected i.p. with the compound FA-6005 (10 mg/kg, which is equal to a final concentration of 100 μM in 5 ml body fluid), and the untreated group (10 mice) was injected with 200 μl of DMSO 1 h prior to inoculating the mice intranasally with 30 LD_50_ (6,000 PFU) of the influenza A/PR/8/34 H1N1 virus in zanamivir, FA-6005, or DMSO. Two doses per day of i.p. zanamivir, FA-6005, or DMSO solutions were given for 7 days. The mice were observed for illness and survival rates for 14 days until death. Five mice from each group were sacrificed at 6 days postinfection to determine viral titers and pathological changes in lungs by a plaque assay (half lung).

### Generation of escape mutant influenza A virus resistance to FA-6005.

The method of generating a mutant virus was performed according to the protocol in our previous study (28). The development of viruses resistant to the selected compound, FA-6005, was performed by serial passage of A/WSN/33 (H1N1) in MDCK cells in the presence of increasing concentrations of inhibitors. The A/WSN/33 (H1N1) parental strain was also passaged in the MDCK cell line without inhibitors as a control. Influenza A/WSN/33 virus at an MOI of 1 was inoculated into MDCK cells with the addition of two concentrations for the compound FA-6005. The lower concentration of compounds used in the first passage was 10 μM, 5-fold higher than the initial EC_50_, while the higher one was 20 μM. At 72 h postinfection, if cytopathic effect (CPE) was observed with both concentrations of the compound, the virus supernatant in the presence of the higher compound concentration would be harvested; otherwise, the one with the lower compound concentration would be harvested. The strategy and the conditions of the compound to generate escape mutants are shown in Table 3.

**TABLE 3:** Strategy used for generating resistant mutant viruses for FA-6005

Virus passage	Concn of FA-6005 (μM)^*a*^
1	10, 20
2	10, 20
3	10, 20
4	20, 40
5	40, 80
6 (plaque purification)	80

a Underlined values signify the inhibitor concentration at which the virus was harvested for escape mutant selection.

As CPE was observed under conditions of a higher concentration of the compound in the first four passages, the concentration of the compound subsequently increased until 80 μM. The desired resistant viral clones were purified by plaque isolation on MDCK cell monolayers in the presence of FA-6005. Three individual purified clones were propagated in MDCK cells in the presence of the compounds, followed by viral RNA extraction, and cDNA of all eight segments was obtained by reverse transcription (RT) using Superscript III reverse transcriptase (Invitrogen), amplified by PCR, and subjected to whole-genome sequencing.

### Generation of recombinant influenza virus by reverse genetics.

The desired recombinant viruses were generated by using the pHW2000 eight-plasmid system (31). The eight plasmids contain the cDNA of the eight segments of the influenza A/WSN/33 (H1N1) genome: pHW2000-PB2, pHW2000-PB1, pHW2000-PA, pHW2000-HA, pHW2000-NP, pHW2000-NA, pHW2000-M, and pHW2000-NS. The influenza virus rescue plasmid was constructed by introducing mutations into the parental plasmid using site-directed mutagenesis. TransLT-Oligo transfection reagent (Mirus) was used to transfect the cocultured 293T and MDCK cells according to the manufacturer’s instructions. The infectious particles from the supernatants were harvested at 72 h posttransfection, and the recombinant virus titer was determined by a plaque assay.

### Growth kinetics of recombinant influenza virus.

MDCK cells were infected by resistant mutant viruses, recombinant virus, or A/WSN/33 (H1N1) virus at an MOI of 0.001. The virus supernatant was collected at 16 h, 24 h, 40 h, 48 h, and 71 h postinfection by high-speed centrifugation to remove cell debris. The collected supernatant was immediately stored at −80°C. Finally, the viral titer of each virus at various time points was measured by a plaque assay to determine its growth rate during the viral life cycle. Each time point was performed in triplicates as described previously (46).

### Protein expression and purification.

Full-length genomic segments of the influenza A/WSN/33 virus NP sequence were cloned into a pET28 vector, and the recombinant plasmid pET28-NP was transformed into competent Escherichia coli strain Rosetta 2. The bacteria were cultured at 37°C until the optical density at 600 nm (OD_600_) of the cell culture reached 0.6. Next, isopropyl-β-thiogalactoside (IPTG) was added to induce protein expression at 18°C for about 16 h. The cell pellet was harvested and lysed by sonication in 50 ml of lysis buffer (20 mM HEPES [pH 7.5], 500 mM NaCl). The cell debris was removed by centrifugation, and the cleared lysate was loaded into a HisTrap HP column (GE Healthcare) mounted on an Äkta purifier (GE Healthcare). The column was washed with 50 ml of lysis buffer, and the protein was then eluted with a linear gradient of 20 to 200 mM imidazole. The peak fractions were collected, concentrated, and loaded onto a Superdex-200 HiLoad 16/60 gel filtration column (GE Healthcare) preequilibrated with buffer containing 20 mM HEPES at pH 7.5 and 150 mM NaCl. The purified protein was concentrated to 20 mg/ml for crystallization.

### Crystallization of FA-6005 with NP.

NP was mixed with FA-6005 (final concentration of 0.5 mM) before crystallization. Crystals were obtained in a solution containing 0.1 M sodium acetate, 0.05 M magnesium acetate, 0.1 M morpholineethanesulfonic acid (MES) (pH 6.0), 7% polyethylene glycol 8000 (PEG 8000), and 5% glycerol, and the crystals were then frozen by quick immersion in liquid nitrogen. X-ray diffraction data were collected at beamline BL17U, Shanghai Synchrotron Radiation Facility, and processed with the HKL2000 package (49). Molecular replacement was applied to solve the phase problem of the NP/FA-6005 complex using the program Phaser (50) in the CCP4 program suite (51). The apo-H1N1 NP monomer structure (Protein Data Bank [PDB] accession number 2IQH (52), chain A) was used as a search model to determine the NP structure, and its tail loop was not included in the initial calculations. The unliganded structural model was refined using the program REFMAC5 (53) in the CCP4 suite, and the program COOT (54) was used for manual model building and adjustment after each refinement cycle of REFMAC5. The space group is C222_1_, with one trimer and three FA-6005 molecules contained in each asymmetric unit, and these three FA-6005 molecules were located in three different sites of the NP molecules. The quality of the current structural model was evaluated by PROCHECK (55) in the CCP4 program suite. Data collection and refinement statistics are summarized in Table 1.

### Time-of-addition assay.

The protocol for the time-of-addition (TOA) assay was performed according to the method described previously by Furuta et al. (47). A total of 5 × 10^4^ MDCK cells per well were cultured 1 day prior to the assay. After 16 to 24 h, cells were washed once with PBS and subjected to A/WSN/33 (H1N1) virus infection at an MOI of 1 on ice (approximately 4°C) in fresh DMEM containing 0.2% FBS and 1 μg/ml TPCK-treated trypsin. After adsorption for 60 min (−1 h), cells were washed three times with PBS, the medium was replaced with fresh DMEM containing 0.2% FBS and 1 μg/ml TPCK-treated trypsin, and the cells were then incubated at 37°C with 5% CO_2_ (time zero). A total of 20 μM FA-6005 or DMSO was added at the indicated time points (−1 to 0, 0 to 2, 2 to 4, 4 to 6, 6 to 8, and 8 to 10 h). After each incubation period, the monolayers with compounds or the control were washed three times with PBS and incubated with fresh medium until 10 h postinfection. Samples were collected at 10 h postinfection and subjected to titer determination using plaque assays after two freeze-and-thaw cycles. Each time point in the TOA assay was conducted in triplicates in 24-well tissue culture plates.

### Luciferase reporter assay for polymerase complex activity.

The luciferase reporter assay was performed as described previously (28). A total of 2 × 10^4^ HEK293T cells per well were seeded in a 96-well tissue culture plate 1 day before the assay. Components of the RNP complex, comprising pHW2000-NP, plus pHW2000-PA, pHW2000-PB1, and pHW2000-PB2, or the respective mutant plasmids, combined with a luciferase reporter plasmid, pHY-Luci, which contains noncoding sequences from the M segment of influenza A virus driven by polymerase I (Pol I), were cotransfected into cultured HEK293T cells using Trans-LT1 transfection reagent (Mirus, Madison, WI) according to the manufacturer’s instructions. phRL-TK (Promega Co., Madison, WI, USA), which expresses renilla luciferase, was also cotransfected as an internal control for data normalization. After 2 h posttransfection, serially diluted compound solutions in 2-fold dilutions were added to the transfected cells. At 24 h posttransfection, cell lysates were prepared for the dual-luciferase reporter assay (Promega Co., Madison, WI, USA), and luciferase activity was measured using a DTX880 multimode detector (Beckman, USA).

### Western blotting.

Cells were infected with influenza A/WSN/33 (H1N1) virus at an MOI of 10 in fresh MEM containing 0.2% FBS and 1 μg/ml TPCK-treated trypsin in the presence of 20 μM FA-6005 or DMSO. At 2, 4, 6, and 8 h postinfection, virus was removed, and cells were washed with PBS once and lysed. Supernatants were collected after centrifugation at 12,000 rpm for 10 min. Equal amounts of proteins dissolved in the cell lysate were loaded into wells of the SDS-PAGE gel, along with a molecular weight marker, and run for 1 to 2 h at 100 V. Gels were transferred to a nitrocellulose membrane at a constant current of 400 mA for 2.5 h. Next, the membrane was blocked with 10% (wt/vol) nonfat milk in PBS plus Tween (PBST) for 1 h at room temperature. Membranes were incubated with the appropriate dilutions of primary antibodies of anti-H1N1 and GAPDH in a blocking buffer overnight and then washed with PBST three times for 15 min each. The membranes were incubated with the recommended dilutions of conjugated secondary antibodies in a blocking buffer at room temperature for 1 h. The membrane was washed with three washes of TBST for 15 min each, dried, and processed for imaging by the Odyssey imaging system (Li-Cor Biosciences, USA).

### Quantitative RT-PCR assays for viral RNA synthesis.

MDCK cells were infected with influenza A/WSN/33 virus at an MOI of 10 in fresh MEM containing 0.2% FBS and 1 μg/ml TPCK-treated trypsin in the presence of 20 μM FA-6005 or DMSO. At 2, 4, 6, and 8 h postinfection, cells were harvested, followed by the extraction of viral RNAs using the RNeasy minikit (Qiagen, Tokyo, Japan) according to the manufacturer’s instructions. Each time point was conducted in triplicates. The expression levels of viral mRNA, vRNA, and cRNA of the NP and NA segments were quantified as described previously (34).

### Fluorescence *in situ* hybridization and immunofluorescence microscopy.

MDCK cells were grown to 70 to 80% confluence on coverslips. Cells were propagated with influenza A/WSN/33 wild-type virus or mutant viruses at an MOI of 10 in fresh MEM containing 0.2% FBS and 1 μg/ml TPCK-treated trypsin. A total of 20 μM FA-6005 or DMSO was added at the indicated time points. Infections were stopped at the indicated time points by fixation with 500 μl of 4% paraformaldehyde (Electron Microscopy Sciences) at room temperature for 15 min. After fixation, the cells were washed with PBS three times and permeabilized with 1 ml 70% ethanol at 4°C overnight. Custom Stellaris fluorescence *in situ* hybridization (FISH) probes for the detection of vRNA and mRNA of segment 5 of influenza virus were designed by utilizing the Stellaris FISH probe designer (Biosearch Technologies Inc., Petaluma, CA). The assay for simultaneous immunofluorescence (IF) microscopy and FISH was performed according to the manufacturer’s instructions (available at www.biosearchtech.com/stellarisprotocols). The slides were stored at 4°C overnight before being sealed with nail polish to prevent drying. The slides were observed and photographed using a Leica DMIL inverted microscope equipped with a DC300F digital imaging system (Leica Microsystems, Germany) or an LSM710 confocal microscope (Carl Zeiss AG, Oberkochen, Germany).

### Pseudotype virus platform.

For the preparation of pseudotype virus, avian pseudotype viruses expressing H5 HA and N1 neuraminidase (NA) were produced by cotransfecting 18 μg of the pNL4-R^−^E^−^Luc^+^ plasmid and 3 μg each of two plasmids (pPolII-HA and pPolII-NA) into 293FT cells with polyethylenimine (PEI) transfection solution (Mirus, Madison, WI). The virus supernatant was collected at 48 h posttransfection by centrifugation to remove the floating cells and cell debris. The supernatant was immediately divided into aliquots and stored at −80°C. Pseudotype virus bearing vesicular stomatitis virus (VSV) envelope protein was also prepared similarly by cotransfecting pNL4-R^−^E^−^Luc^+^ and pVSV plasmids. For the antiviral assay for pseudotype virus, the compound was 2-fold serially diluted in DMEM supplemented with 10% FBS and then incubated with 100 50% tissue culture infective doses (TCID_50_) of pseudotype virus at room temperature for 1 h. MDCK cell monolayers were infected with the compound-virus mixture at 37°C for 72 h. Finally, cells were lysed, and the luciferase reading was recorded using a luciferase assay system (Promega) according to the manufacturer’s manual. The luciferase reading of cells treated with the compound FA-6005 only was regarded as the background, while the samples incubated with the virus and DMSO were regarded as representing maximum transduction. The EC_50_ of FA-6005 against the pseudotype virus was estimated by determining the percentage of inhibition.

### Uncoating assay and RNP import assay.

Confluent MDCK cells were infected with influenza A WSN/33 virus or a corresponding mutant virus in an infection medium (MEM with 0.2% FBS and 1 μg TPCK-treated trypsin) at an MOI of 100 at 4°C for 1 h. After virus adsorption, the cells were washed with ice-cold PBS to remove the unbound virus particles. Fresh infection medium, with or without compounds, was added to the bound particles, and cells were allowed to internalize at 37°C with a 5% CO_2_ incubator at the indicated times. A total of 1 mM cycloheximide was added to the infection medium to prevent the synthesis of new viral proteins. Following 2 h of infection, the infected cells were fixed and permeabilized with 0.1% Triton X-100 in PBS for 3 to 5 min at RT and incubated with purified mouse monoclonal M1 antibody (Abcam, USA) in PBS (1:100) for 1 h to stain the viral M1. Cells were washed with PBS, followed by incubation with secondary anti-mouse IgG-AF488 (1:150). Nuclei were stained with ProLong gold antifade reagent with 4′,6-diamidino-2-phenylindole (DAPI; Thermo Fisher Scientific, USA). Coverslips were then imaged. While RNP import was detected at 3 h of infection, the infected cells were stained with purified mouse monoclonal NP antibodies in PBS (1:150) for 1 h to stain the viral RNP, followed by incubation with secondary anti-mouse IgG-AF488 (1:150). Nuclei were stained with ProLong gold antifade reagent with DAPI (Thermo Fisher Scientific, USA). Coverslips were then imaged.

### Pulldown assays.

The protocol for the pulldown of FLAG-tagged proteins was modified and performed using anti-FLAG M2 magnetic beads (Sigma-Aldrich Inc., St. Louis, MO, USA) as previously described by M. J. Amorim et al. (24). Confluent 6-well dishes of MDCK cells were transfected with 500 ng of pHW2000-FLAG-GFP-NP. After 24 to 48 h, cells were infected with influenza A WSN/33 virus at an MOI equal to 10. At 6 h postinfection, 20 μM FA-6005 was added to the infected cells. DMSO was added as a negative control. All samples were collected and lysed at 8 h p.i. in 500 μl of a solution containing 50 mM Tris-HCl (pH 7.4), 150 mM NaCl, 1% Triton X-100, and a protease inhibitor cocktail (Roche Life Science, Switzerland) on ice for 30 min. Cell lysates were precleared with mouse IgG agarose, and the supernatant was bound to mouse anti-FLAG M2 magnetic beads on ice for 2 h. Beads were washed before and after sample binding with a wash buffer containing 50 mM NaCl, 50 mM Tris (pH 7.5), 1 mM EDTA, 0.5% NP-40, and 10% glycerol (Sigma-Aldrich Inc., St. Louis, MO, USA). Bound proteins were eluted by being boiled in SDS-PAGE sample buffer. Western blotting was performed and imaged as described above.

### Electrophoretic mobility shift assay.

NPs were incubated with compounds at RT for 30 min, followed by the addition of 24-nucleotide (nt) small RNA, incubation for another 30 min, and the addition of up to 10 μl of nuclease-free water. The final concentration of small RNA was 2 μM, and the molar ratio of NP to RNA was kept at 4:1 (18). After incubation, the samples were mixed with 3 μl 6× DNA loading dye (0.25% bromophenol blue, 0.25% xylene cyanol, 40% sucrose) and loaded into sample wells of a nondenatured 4-to-12% gradient Bis-Tris NuPAGE gel (Thermo Fisher Scientific, USA). The gel was equilibrated by preelectrophoresis at 50 V in 1× Tris-borate-EDTA (TBE), and electrophoresis was performed at a constant voltage of 150 V for 35 min at room temperature in 1× TBE. The gel was visualized by EtBr staining for small RNA shift patterns. The same gel was also stained with Coomassie brilliant blue G-250 to display NP shift patterns.

### Transmission electron microscopy.

To understand the effects of the compound on virion formation, transmission electron microscopy (TEM) was performed as described in our previous study (17). A total of 5 × 10^6^ MDCK cells were seeded into a 10-mm tissue culture dish (TPP, Switzerland) in MEM with 10% FBS. On the following day, cells were washed once with PBS and infected with influenza A/WSN/33 or corresponding mutant viruses at an MOI of 10 in MEM with 0.2% FBS and 1 μg/ml TPCK-treated trypsin. Budding virions of either strain were not detected at 4 h p.i. as we described previously; thus, 20 μM compound was added to the cells after 6 h p.i., and DMSO was added as a control. At 8 h postinfection, cells were washed with 0.1 M phosphate buffer at pH 7.4 three times and incubated at 4°C in 2.5% glutaraldehyde (electron microscopy grade) in 0.1 M phosphate buffer (pH 7.4) overnight. The monolayer was washed with phosphate buffer the following day, and cells were scraped into phosphate buffer and harvested by centrifugation at 14,000 × *g* for 10 min. The supernatant was replaced with fresh phosphate buffer and processed by the Electron Microscope Unit at The University of Hong Kong (HKU). Images were acquired using an FEI Philips CM100 transmission electron microscope equipped with a Deben AMT digital camera and an Edax Genesis XM4 EDX system.

### Statistical analysis.

Statistical analysis was performed using GraphPad Prism. Statistical significance was determined with Student’s *t* test and two-way analysis of variance (ANOVA). *P* values of <0.05 were considered significant (*, *P < *0.05; **, *P < *0.01; ***, *P < *0.001; ****, *P < *0.0001; ns, no statistical significance).

### Data availability.

Coordinates and structure factors have been deposited in the PDB with accession number 6J1U.
